# Age Assessment of Youth and Young Adults Using Magnetic Resonance Imaging of the Knee: A Deep Learning Approach

**DOI:** 10.2196/16291

**Published:** 2019-12-05

**Authors:** Ana Luiza Dallora, Johan Sanmartin Berglund, Martin Brogren, Ola Kvist, Sandra Diaz Ruiz, André Dübbel, Peter Anderberg

**Affiliations:** 1 Department of Health Blekinge Institute of Technology Karlskrona Sweden; 2 Optriva AB Stockholm Sweden; 3 Department of Pediatric Radiology Karolinska University Hospital Stockholm Sweden

**Keywords:** age assessment, bone age, skeletal maturity, deep learning, convolutional neural networks, transfer learning, machine learning, magnetic resonance imaging, medical imaging, knee

## Abstract

**Background:**

Bone age assessment (BAA) is an important tool for diagnosis and in determining the time of treatment in a number of pediatric clinical scenarios, as well as in legal settings where it is used to estimate the chronological age of an individual where valid documents are lacking. Traditional methods for BAA suffer from drawbacks, such as exposing juveniles to radiation, intra- and interrater variability, and the time spent on the assessment. The employment of automated methods such as deep learning and the use of magnetic resonance imaging (MRI) can address these drawbacks and improve the assessment of age.

**Objective:**

The aim of this paper is to propose an automated approach for age assessment of youth and young adults in the age range when the length growth ceases and growth zones are closed (14-21 years of age) by employing deep learning using MRI of the knee.

**Methods:**

This study carried out MRI examinations of the knee of 402 volunteer subjects—221 males (55.0%) and 181 (45.0%) females—aged 14-21 years. The method comprised two convolutional neural network (CNN) models: the first one selected the most informative images of an MRI sequence, concerning age-assessment purposes; these were then used in the second module, which was responsible for the age estimation. Different CNN architectures were tested, both training from scratch and employing transfer learning.

**Results:**

The CNN architecture that provided the best results was GoogLeNet pretrained on the ImageNet database. The proposed method was able to assess the age of male subjects in the range of 14-20.5 years, with a mean absolute error (MAE) of 0.793 years, and of female subjects in the range of 14-19.5 years, with an MAE of 0.988 years. Regarding the classification of minors—with the threshold of 18 years of age—an accuracy of 98.1% for male subjects and 95.0% for female subjects was achieved.

**Conclusions:**

The proposed method was able to assess the age of youth and young adults from 14 to 20.5 years of age for male subjects and 14 to 19.5 years of age for female subjects in a fully automated manner, without the use of ionizing radiation, addressing the drawbacks of traditional methods.

## Introduction

### Background

Bone age and skeletal maturity are closely related concepts that measure the stage of bone development of an individual [1,2]. When compared to the chronological age, they aid in the diagnosis and in determining the time of treatment of many pediatric disorders related to orthodontics, orthopedics, and endocrinology. Further, they are also used in estimations about the final height of an individual [3].

From a legal standpoint, bone age assessment (BAA) also plays an important role in the estimation of chronological age. In this sense, the estimation of the bone age is employed when determining if an individual is a minor in the absence of valid documents, which is the case for numerous unaccompanied minors seeking asylum [2], as well as in adoption, imputability, and pedopornography judicial and civil issues [4]. The estimation of chronological age is also used in age-related sports competitions to guarantee fair play [5,6]. In all of these cases, BAA is an important tool that is used to make important legal decisions that can enormously affect an individual's life.

The traditional methods for performing BAA are the Greulich-Pyle (GP) atlas and the Tanner-Whitehouse (TW) scoring system. The GP atlas [7] comprises hand and wrist radiograph reference images of subjects from 0 to 19 years of age for males and 0 to 18 years of age for females. The process for determining bone age is done by comparing the nearest matching reference image in the atlas to the image of the individual being assessed [3]. The TW scoring system [8] first analyzes the hand and wrist radiograph of a subject and categorizes the skeletal maturity scores of the ossification centers of the radius, ulna, and 13 short bones of the hand and carpals into stages ranging from A to I. Then, all of the stages are aggregated into a numerical score that is converted to the bone age [2].

### Drawbacks of the Traditional Age-Assessment Methods

The drawbacks of the GP and TW methods derive from the fact that they are done manually by radiologists; thus, they can be prone to inter- and intrarater variability, in addition to being time-consuming tasks [9,10].

Also, there is an important ethical issue related to submitting healthy subjects to ionizing radiation without therapeutic purposes, which is especially important in the case of assessing if an individual is a minor for legal purposes [10]. This scenario suggests that new approaches for the assessment of age should be explored by research in order to address these drawbacks.

The use of radiation-free medical imaging can be achieved by the employment of magnetic resonance imaging (MRI). An additional advantage of MRI technology is that it supports the manipulation of the image's contrast, granting the possibility of highlighting different tissue types and allowing better visualization of ossification centers [11,12]. Additionally, since MRI images are volumetric, more information can be extracted and analyzed when compared to 2D radiographs [13].

The issues related to rater variability and time spent in the assessment are big motivators for the use of more automated techniques like deep learning. Deep learning is a type of machine learning technique, which refers to algorithms that are able to learn a task from a set of training examples; in view of a new set of data, this task can be reproduced with an acceptable performance [14]. The use of machine learning for health applications is not new and is broadly employed for disease prediction and prognosis [14,15], genomics, proteomics, and microarrays [16]; it has also been used to predict health care utilization through Web search logs [17]. Contrary to many machine learning techniques, deep learning methods perform feature engineering: instead of having a domain expert specify important data characteristics, it learns the informative representations in the data and performs a task of classification or regression [18,19]. When working with medical images, this is especially advantageous since image features are difficult to translate into descriptive means [20]. That is the reason why the first applications of deep learning with health data were aimed at analyzing medical images, specifically MRI images of the brain for the prediction of Alzheimer disease and MRI images of the knee to estimate the risk of osteoarthritis [21]. In the specific area of BAA, most computerized approaches extract features following established procedures (eg, TW or GP), which can be limiting in terms of the information available in the image [22]. When using deep learning, the algorithm finds the important representations in the images without any constraint, which could allow more features in the image to be considered in the classification or regression task not previously known by the current methods [22].

### Goal of This Study

Taking into account the numerous settings in which the estimation of chronological age is employed and their importance and potential effect on individuals' lives, it is important to address the drawbacks in the methods currently in use. Thus, this paper proposes an automated approach for age assessment of youth and young adults (14-21 years of age) employing deep learning methods with MRI images of the knee.

The knee region aggregates four ossification centers—femur, tibia, fibula, and patella—but it has not been explored very much by the research in BAA, which is mostly focused on the hand and wrist regions; this research makes use of radiograph images, due to the impact the GP method, which is still considered by many to be the gold standard for BAA [23]. The choice of the knee region in this study was motivated by findings in the research with MRI images that reported the presence of cartilage signal intensity at the knee ossification centers in male individuals from 17.8 to 30.0 years of age and female individuals from 16.6 to 29.6 years of age, which could imply later fusion of maturation centers [24]. Additionally, recent findings in the research of BAA with MRI images of the knee also reported a uniform spatial pattern of maturation of ossification centers in the knee in both male and female individuals [12].

## Methods

### Overview

The fully automated age-assessment method proposed in this paper uses MRI images of the knee and the subjects' chronological ages to train deep learning models for continuous age estimation with convolutional neural networks (CNNs).

**Figure 1:**
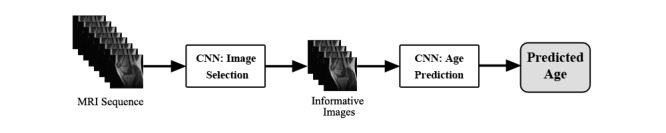
Overview of the proposed automated age-assessment method. CNN: convolutional neural network; MRI: magnetic resonance imaging.

An overview of the method is shown in Figure 1. It comprises two CNN models: the first one is responsible for selecting the most informative images of an MRI sequence for age-assessment purposes; these are then fed to the age-prediction CNN, which outputs an estimated age. The remainder of this section further details the process of training, deploying, and evaluating the CNN models of the proposed method as well as the materials used in the experiments.

### Recruitment

This study prospectively acquired MRI images of the knee region of 402 volunteer subjects—221 males (55.0%) and 181 (45.0%) females—aged 14.0-21.5 years (see Table 1) between 2017 and 2018. It is important to note that throughout the text of this paper, the mention of an age group X refers to an age span from X to X.5 (eg, the age group 14 refers to an age span of 14 to 14.5 years). The criteria used for subject recruitment in the study were as follows:

1. Inclusion criteria: subjects (1) were born in Sweden and (2) have a birth certificate verified by national authorities.
2. Exclusion criteria: subjects (1) have a history of bilateral fractures or trauma near the growth plate, (2) have a history of chronic disease or long-term medication, (3) exhibit noncompliance during MRI examinations, (4) have resided outside Sweden for more than 6 consecutive months, and (5) experienced a past pregnancy or were pregnant at the time of recruitment: all female volunteer subjects were tested.

**Table 1:** Age distribution of the volunteer subjects^a^ (N=402).

Gender	Subject age group^b^, years, n (%)								Total, n (%)
	14	15	16	17	18	19	20	21	
Male (N=221)	22 (10.0)	26 (11.8)	31 (14.0)	25 (11.3)	24 (10.9)	25 (11.0)	35 (15.8)	33 (14.9)	221 (100)
Female (N=181)	22 (12.2)	21 (11.6)	30 (16.6)	27 (14.9)	20 (11.0)	12 (6.6)	25 (13.8)	24 (13.3)	181 (100)
Total (N=402)	44 (10.9)	47 (11.7)	61 (15.2)	52 (12.9)	44 (10.9)	37 (9.2)	60 (14.9)	57 (14.1)	402 (100)

^a^All data were acquired within a maximum of 6 months after the subjects' birth dates.

^b^Age group X refers to an age span from X to X.5 (eg, the age group 14 refers to an age span of 14 to 14.5 years).

### Magnetic Resonance Imaging Examinations

The MRI examinations were performed on 1.5 Tesla whole-body MRI scanners with dedicated knee coils. The images were taken from the nondominant side of the knee; however, in the case of previous fracture or trauma near these regions, the dominant side was imaged.

The examinations were performed in two sites, with the same protocol, 256 x 256-pixel resolution, and 160 x 160 mm field of view. The following machinery was used:

1. Site 1: MAGNETOM Avanto Fit (Siemens Healthcare Gmbh) and Achieva (Philips Healthcare) whole-body scanners.
2. Site 2: SIGNA (GE Healthcare) whole-body scanner.

### Data Privacy and Study Ethics

All acquired data were anonymized and stratified by age and gender. The study was approved by the local ethics committee and was conducted in accordance with the Declaration of Helsinki. Written informed consent was acquired from all subjects and legal guardians, in the case of minors.

### Image Selection

Each MRI examination produced 17-35 images per subject, however, not all of them were equally informative in regard to the assessment of the age of an individual. To simplify the age estimation learning task, only the best images were considered for the *CNN: Age Prediction* model. To make the method fully automated without any need for human input, a CNN classifier was trained to be able to select the most informative images in an MRI sequence. An *informative* image in the context of the proposed method corresponds to the part of the bone that contains anatomical structures of interest, which include the growth plate, epiphysis, and metaphysis. This classifier corresponds to the *CNN: Image Selection* block in Figure 1.

The CNN architecture used was GoogLeNet [25], a model that has been shown to generalize well to a wide variety of image classification tasks, medical and otherwise [26].

**Figure 2:**
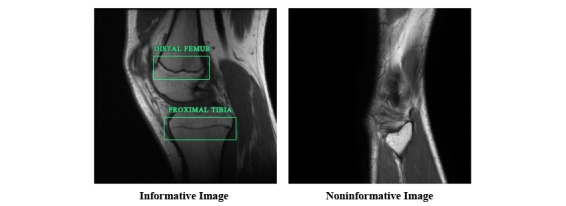
Examples of informative and noninformative images from the same subject.

To be able to train this classifier, one image from each MRI sequence that had growth zones clearly visible was annotated as *informative*. Also, one image from each MRI sequence in which the growth zones were occluded by other tissue types was selected and labelled as *noninformative*. Examples of informative and noninformative images are shown in Figure 2.

The output of the CNN model is the confidence levels of the two classes—informative and noninformative—for the given MRI image. The confidence level is a continuous value between 0 and 1, where 1 is the highest confidence level and the confidence levels of the two classes sum up to 1. In later steps, only images with a confidence level for the informative class above a threshold C on the test set were used.

### Age Prediction

For predicting the age of an individual from the MRI images, another CNN model was built. This model corresponds to the *CNN: Age Prediction* block in Figure 1. Seven different CNN architectures were considered; these were as follows: GoogLeNet [25], ResNet-50 [27], Inception-v3 [28], Visual Geometry Group (VGG) [29], AlexNet [30], DenseNet [31], and U-Net [32].

The final classification layer of these networks was replaced with a linear scalar output providing the age estimation. The only exception from this was U-Net, which is a fully connected model without classification layers in the end. Here, the linear scalar output was added after the last convolutional layer instead.

**Figure 3:**
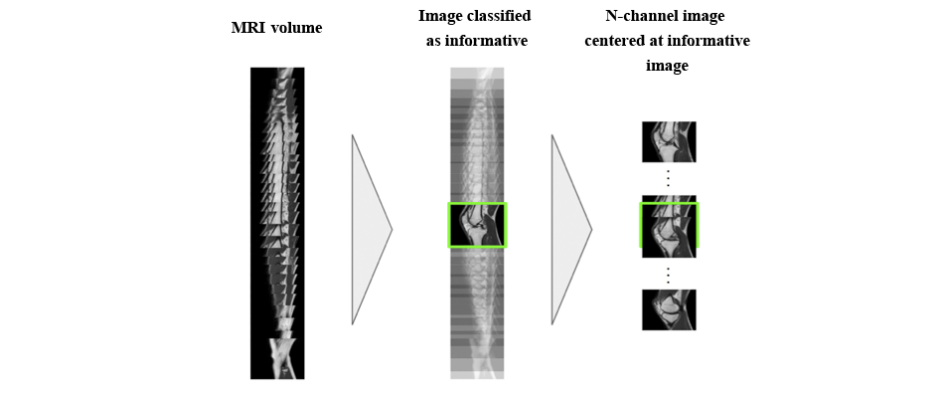
Example of how an N-channel image is created from one of the images in the magnetic resonance imaging (MRI) volume classified as informative.

The age-prediction model takes an MRI image with N channels as input, then outputs the estimated chronological age of the subject. To create an image with N channels, a subset of the MRI volume, centered on an image classified as informative, is extracted (see Figure 3).

Input images of 1-9 channels were tested. The idea was that the model might be able to use information from neighboring images to improve results and make the model more robust to mistakes in the image-selection process.

### Training the Models

#### Training and Evaluation

The Convolutional Architecture for Fast Feature Embedding (Caffe) deep learning framework [33] was used to train the models. Training and evaluation were done on Amazon Web Services on an Elastic Compute Cloud (EC2) P3.2xlarge with a Tesla V100 Nvidia graphics processing unit.

#### Optimization

The Adam optimizer [34] was used to minimize the cross-entropy loss when training the classifier and the Euclidean loss when training the regressor. Cross-entropy loss for binary classification is calculated as follows:

–1/*N* Σ*^N^_i_*_=1_*y_i_* × *log*(*p*(*y_i_*)) + (1–*y_i_*) × *log*(1–*p*(*y_i_*)) (1)

with *N* being the number of training samples per batch, *y* being a binary indicator (0 or 1) of the correctness of classification for an observation *o* being of class *c*, and *p* being the predicted probability of an observation *o* being of class *c*. Euclidean loss is calculated as follows:

1/2*N* Σ*^N^_i_*_=1_ │|*x_i_*^1^=*x_i_*^2^|│ ^2^_2_ (2)

with *N* being the number of training samples per batch, *x*^1^ the estimated age, and *x*^2^ the verified chronological age.

#### Cross-Validation

All experiments were performed using six-fold cross-validation, including the test set. The dataset was split into six equal-sized parts, with data stratified for age and gender. This data partition followed the procedure that all of the images from a subject were assigned to a single fold. Four parts were used for training, one part was used for validation during training, and one part was used to finally evaluate and measure the model’s performance. This was done to be able to evaluate the models on the full dataset.

Before performing a full cross-validation, a sparse grid search was performed for each model to find good hyperparameters. This was done using the validation set of the first cross-validation split only. The hyperparameters tuned during the grid search were as follows: learning rate, weight decay, momentum, dropout ratio, and batch size.

#### Transfer Learning

Both training from scratch and transfer learning were tested. Transfer learning is a technique that, instead of using randomly initialized weights, takes the weights from a CNN that has already been trained to perform well on a generic task as a starting point. The model is then adapted by carefully updating the weights using the task-specific training data. This makes it possible to leverage larger datasets to avoid overfitting when the task-specific dataset is small [35,36]. All pretrained models used in this paper were trained on ImageNet [37]. During the task-specific training, the weights of all layers were updated.

#### Data Augmentation

Data augmentation is a technique that aims to synthetically increase the size of the training set from existing data without additional labelling work, using geometric or photometric transformations, noise injections, and color jittering operations. It is used to prevent overfitting when training CNNs on small datasets [38,39].

**Figure 4:**
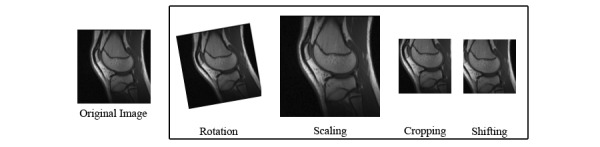
Examples of data augmentation operations applied in the proposed method.

In the proposed method, data augmentation was performed on all training samples to increase the dataset. The images were randomly cropped, shifted, rotated at a maximum of five degrees, and scaled up to 20%. Figure 4 shows examples of the applied data augmentation operations.

### Estimation

When estimating the age on the test set for each subject, all images with a confidence higher than threshold C of 0.95 for the informative class were used. Each of these test images were used to create a number of copies with different augmentations applied to each copy. All augmented test images were fed through the network to produce one result each. Finally, the results from the augmented versions of the images were used to estimate a final result. This technique has been shown to improve the performance of the predictions and is widely used within deep learning [25].

In this method, each image was augmented 15 times, using the same augmentations as during training, generating 15 new images. If none of the images for a subject had a confidence higher than the threshold, the image with the highest confidence was used instead. This was the case for two subjects only. The highest confidence value for these subjects were 0.91 and 0.81. If more than 10 images had a confidence level higher than the threshold, only the 10 images with the highest confidence were used in order to set a maximum limit on the processing time.

Age was estimated for all augmented images and, finally, the median of all estimated ages for each subject was computed to get the final prediction. For example, if a subject had eight images with high-enough confidence, 120 augmented images were created and 120 ages were estimated, of which the median was used as the final estimated age.

## Results

### Overview

Hyperparameters and settings were tuned to optimize the models' performance. This was done through a sparse grid search on the first cross-validation split, as specified previously. The validation set was used for tuning in order to avoid tuning specifically toward the test set and thereby overestimating the models' performance on new data. The final results reported in this section were evaluated on the full dataset from the cross-validation test sets in terms of the mean absolute error (MAE), calculated as follows:

MAE = 1/*n* Σ*^n^_i_*_=1_ |*x_i_*–*x*| (3)

with *n* being the number of samples, *x_i_* being the estimated age, and *x* being the verified chronological age.

### Conclusions From Experiments

Fine-tuning pretrained models showed significantly better results compared to training the models from scratch. The two architectures that showed best results were GoogLeNet and ResNet-50. Training on men and women subjects separately gave better results for both groups compared to single training using all data.

The best results were achieved using a confidence threshold C of 0.95 in the image selection data preprocessing stage for choosing the most informative MRI images. The results did not change much using different thresholds. MAE differed only by 0.004 years when using thresholds in the range of 0.5-0.99.

Results were very similar when using MRI images with one or three channels, but with more channels than three the performance dropped. This can be due to the increasing number of parameters in the models when using more channels, which might lead to overfitting. Using one channel gave a slightly better result, which is why we used this in our final models.

The hyperparameters that gave the best results were as follows:

1. Learning rate: 1e-4
2. Weight decay: 1e-2
3. Momentum: 0.83
4. Dropout ratio: 0.7 for GoogLeNet and 0.6 for ResNet-50
5. Batch size: 66 for GoogLeNet and 30 for ResNet-50

The best results were achieved when resizing the images to 256×256 pixels for both GoogLeNet and ResNet-50. Both these architectures use cropped images of size 224×224 pixels as input.

### Results for the Best Models

**Figure 5:**
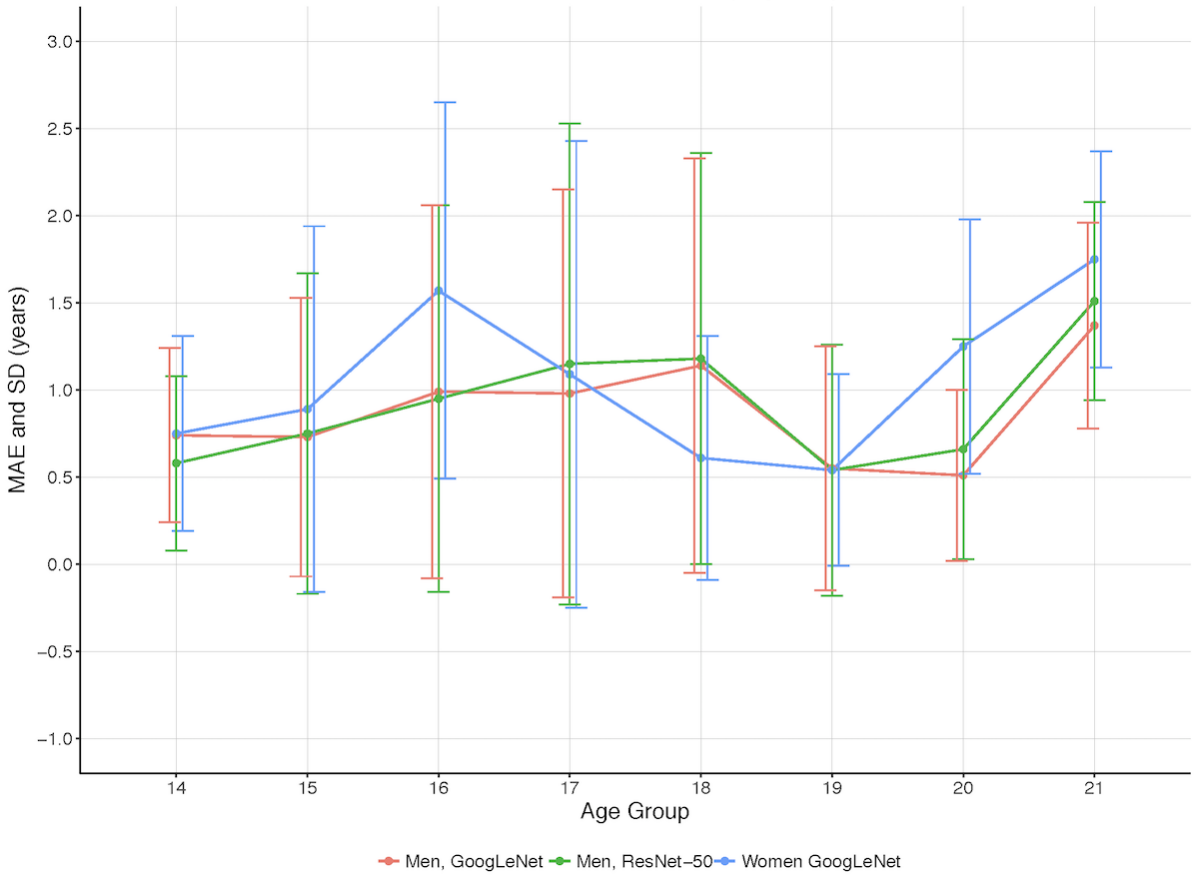
Comparison of the best-performing models: GoogLeNet and ResNet-50. MAE: mean absolute error.

The results for the experiments with the best-performing models, GoogLeNet and ResNet-50, in terms of the MAE and SD per age group is shown in Figure 5 and detailed in Table 2 below. The acquisition of the MRI images happened in a window within 6 months from the subjects' birthdays. The best overall results for male subjects were achieved by the GoogLeNet model using knee MRI images. When training the age-prediction model for women, only the architecture performing best on men was considered.

**Figure 6:**
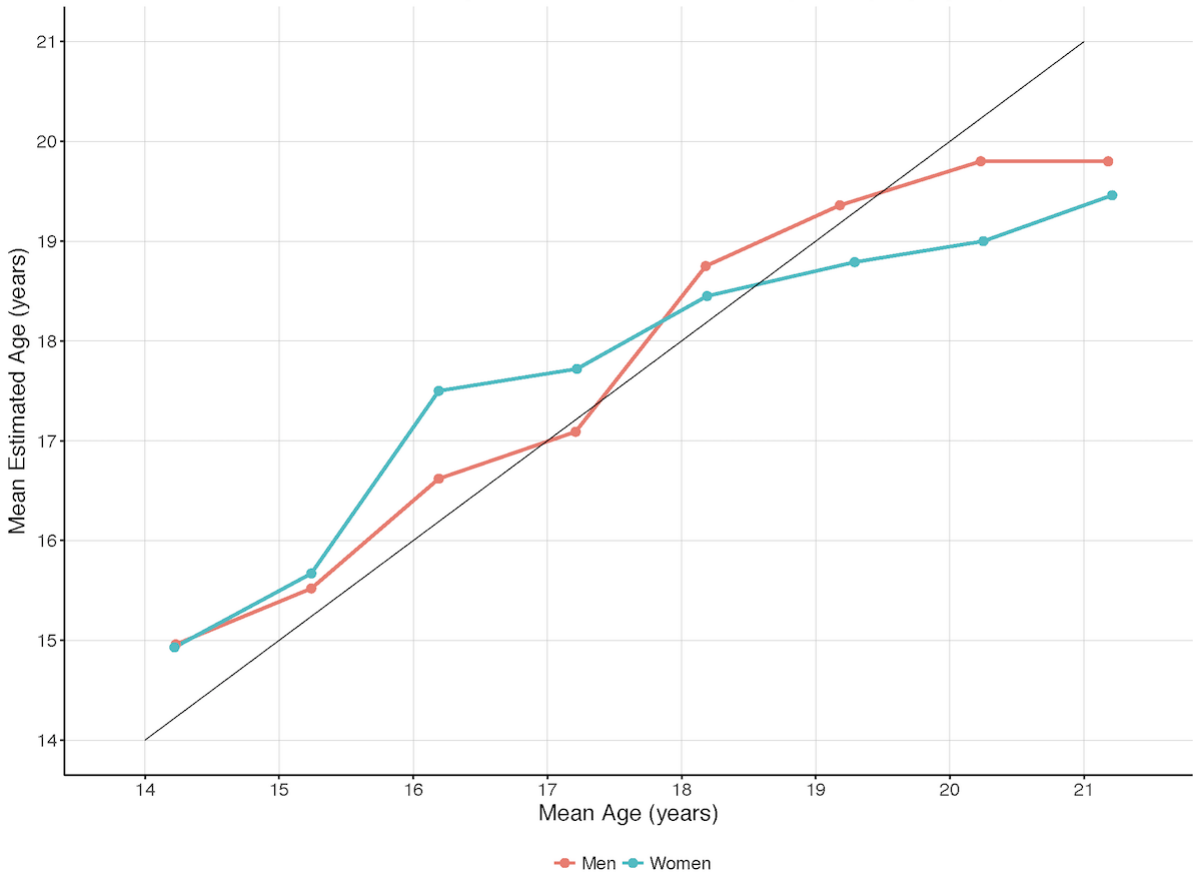
Mean age and mean estimated age per age group with the best-performing model, GoogLeNet, on male and female subjects.

There is a clear trend on all of the experiments among male subjects in which the MAE increases substantially from the age of 21. The same phenomenon occurs for the model among women subjects but from the age of 20. These results lead us to believe that after the ages of 20.5 for men and 19.5 for women, no information regarding older ages can be extracted from the MRI image data, regarding the knee region. This is also supported by Figure 6 and Table 3, which show that the mean estimated age planes out around these ages for the respective genders. The models underestimated the age more and more the older the subjects got after these ages. In conclusion, the presented method is not able to estimate ages above 20.5 for men and above 19.5 for women. Therefore, these ages were removed in the results below, which focus on the applicable age ranges for the models: 14 to 20.5 years for men and 14 to 19.5 years for women.

**Table 2:** Results from the experiments with the best-performing models: GoogLeNet and ResNet-50.

Gender, model	Subject age group^a^ in years, MAE^b^ (SD)							
	14	15	16	17	18	19	20	21
Men, GoogLeNet	0.74 (0.50)	0.73 (0.80)	0.99 (1.07)	0.98 (1.17)	1.14 (1.19)	0.55 (0.70)	0.51 (0.49)	1.37 (0.59)
Men, ResNet-50	0.58 (0.50)	0.75 (0.92)	0.95 (1.11)	1.15 (1.38)	1.18 (1.18)	0.54 (0.72)	0.66 (0.63)	1.51 (0.57)
Women, GoogLeNet	0.75 (0.56)	0.89 (1.05)	1.57 (1.08)	1.09 (1.34)	0.61 (0.70)	0.54 (0.55)	1.25 (0.73)	1.75 (0.62)

^a^Age group X refers to an age span from X to X.5 (eg, the age group 14 refers to an age span of 14 to 14.5 years).

^b^MAE: mean absolute error.

**Table 3:** Mean age and mean estimated age per age group by the best-performing model, GoogLeNet, on male and female subjects.

Gender	Subject age group^a^, years							
	14	15	16	17	18	19	20	21
Men, mean age	14.23	15.24	16.19	17.21	18.18	19.18	20.23	21.18
Men, mean estimated age	14.96	15.52	16.62	17.09	18.75	19.36	19.80	19.80
Women, mean age	14.22	15.24	16.19	17.22	18.19	19.29	20.25	21.21
Women, mean estimated age	14.93	15.67	17.50	17.72	18.45	18.79	19.00	19.00

^a^Age group X refers to an age span from X to X.5 (eg, the age group 14 refers to an age span of 14 to 14.5 years).

### Results for the Best Models in the Applicable Age Ranges

**Figure 7:**
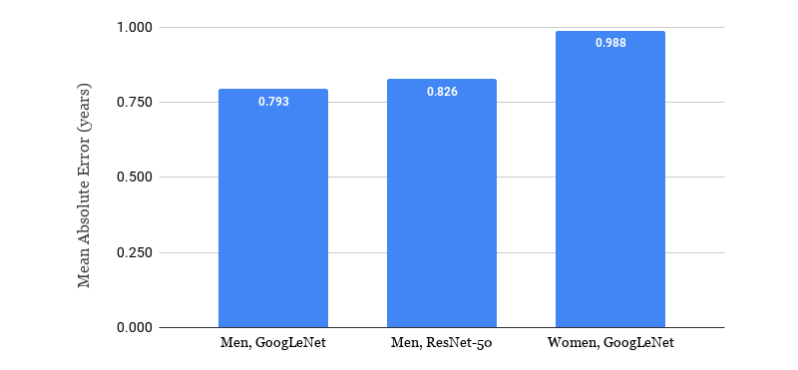
Mean absolute error (MAE) of the best-performing models in the applicable age ranges.

Figure 7 shows the MAE in years for the best models in their applicable ranges: 14-20.5 years for men and 14-19.5 years for women. The best achieved result for the age prediction of youth and young adult individuals in this study corresponds to an MAE of 0.793 years for men and 0.988 years for women, using the GoogleNet architecture.

### Results for the GoogLeNet Model in the Applicable Age Ranges for Male and Female Subjects

**Figure 8:**
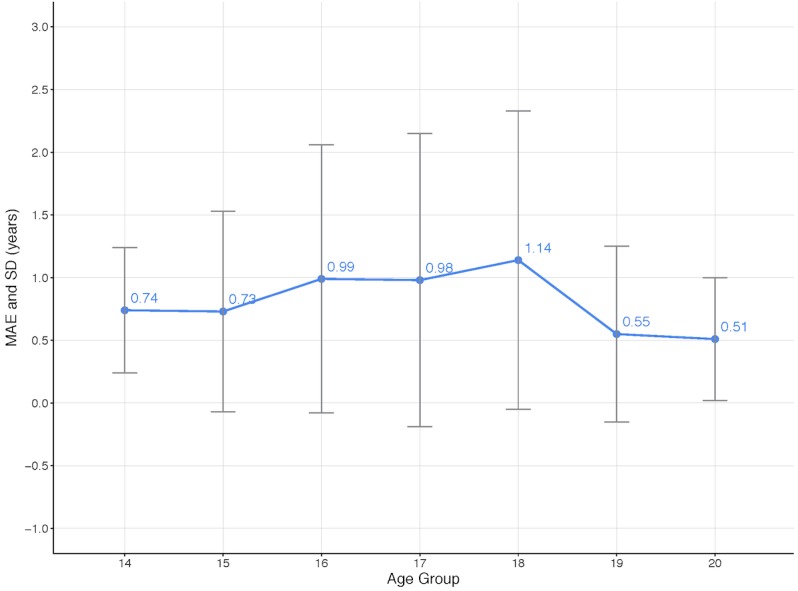
Mean absolute error (MAE) for the GoogLeNet model for male subjects in the applicable age ranges.

**Figure 9:**
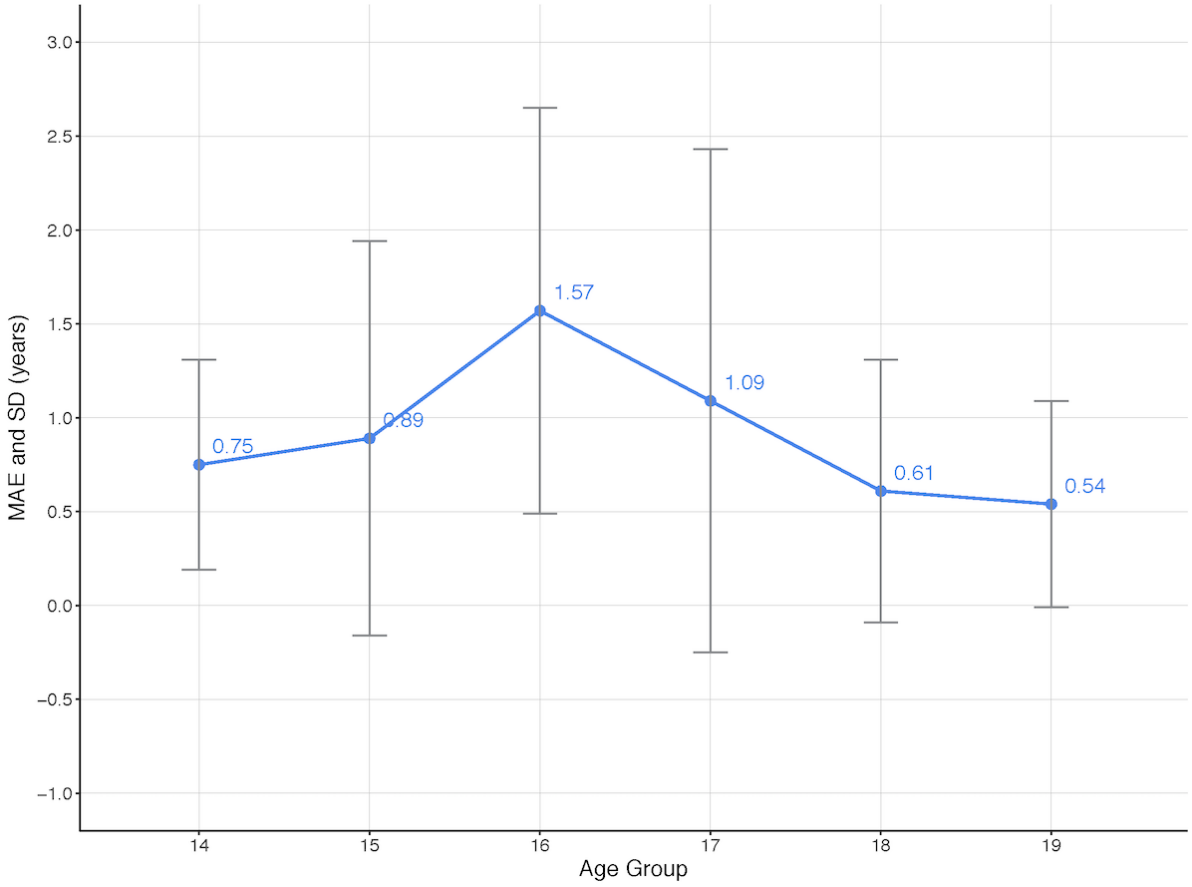
Mean absolute error (MAE) for the GoogLeNet model for female subjects in the applicable age ranges.

Figures 8 and 9 show the MAE for the GoogLeNet model applied to male and female subjects, respectively, in the applicable age ranges. It is interesting to notice that the age range with the highest error occurs earlier for females (age group of 16) compared to men (age group of 18). This goes in line with previous knee studies where findings showed that women mature earlier than men [40].

### Classification Performance of Minors Versus Adults

Experiments were also performed for classification of subjects as being adults or minors, considering the age of 18 years old as the adulthood threshold. This classification is especially important in cases regarding the age assessment of minors from a legal standpoint.

No new training of models was performed. Instead, the classification of adults and minors was performed by applying a threshold to the estimated age from the best-performing models trained in the age-assessment experiments.

Three different strategies for setting the threshold were evaluated:

1. Setting the threshold to increase the accuracy for minors and sacrificing accuracy for adults.
2. Setting the threshold to get as equal accuracy as possible for adults and minors.
3. Using the threshold of 18 years of age without any modification.

**Figure 10:**
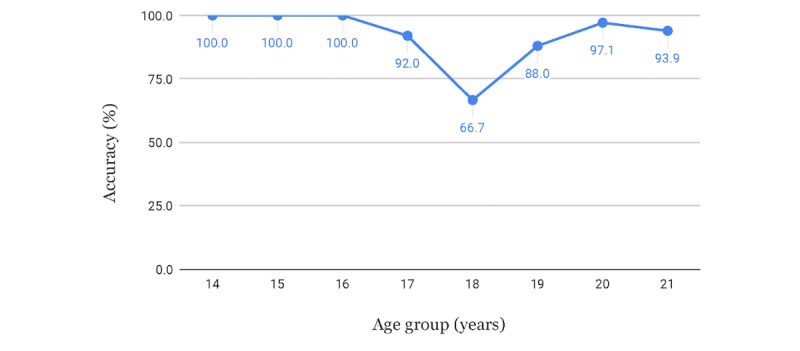
Accuracies for minor versus adult classification of male subjects, using threshold to increase accuracy for minors.

**Figure 11:**
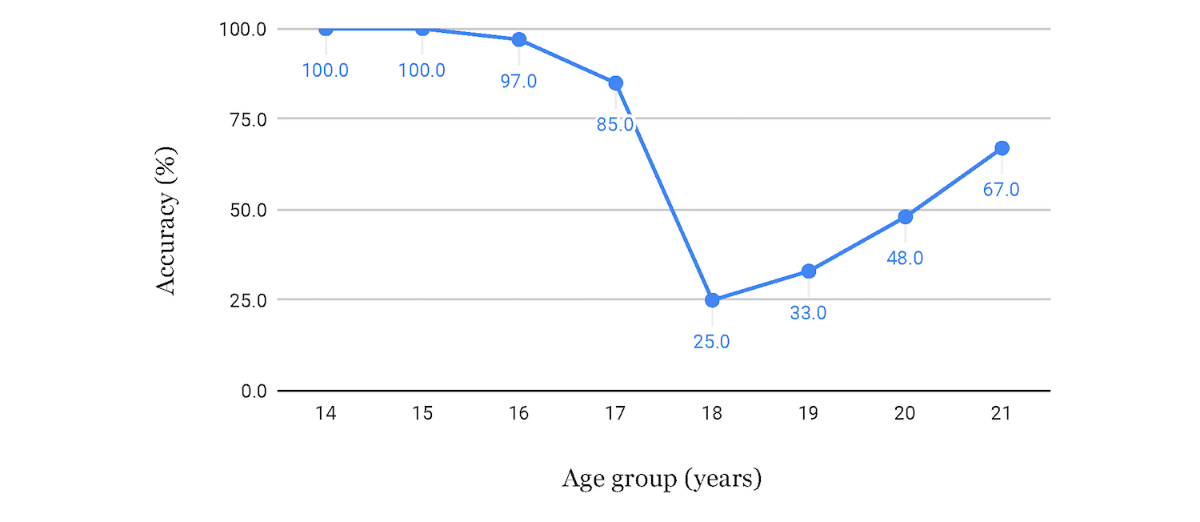
Accuracies for minor versus adult classification of female subjects, using threshold to increase accuracy for minors.

The results for male subjects are shown in Figure 10 and Table 4. The same procedures and reasoning were also applied to the women's case and the results are shown in Figure 11 and Table 5.

**Table 4:** Accuracies for minor versus adult classification of male subjects.

Strategy for setting the threshold	Threshold in years	Accuracy for minors, %	Accuracy for adults, %
Using the threshold to get lower errors for minors	18.73	98.1	88.0
Using the threshold to get as equal accuracy for adults and minors as possible	18.38	93.3	93.2
Using estimated age without modifying the threshold	18.00	90.4	95.7

**Table 5:** Accuracies for minor versus adult classification of female subjects.

Strategy for setting the threshold	Threshold in years	Accuracy for minors, %	Accuracy for adults, %
Using threshold to get lower errors for minors	19.11	95.0	45.7
Using threshold to get as equal accuracy for adults and minors as possible	18.20	85.0	85.2
Using estimated age without modifying the threshold	18.00	77.0	88.9

## Discussion

### Principal Findings

This paper proposed a fully automated method, free from ionizing radiation, for age assessment based on MRI images of the knee using CNNs. The method was able to assess the age of male subjects in the range of 14-20.5 years of age, with an MAE of 0.793 years, and of female subjects in the range of 14-19.5 years of age, with an MAE of 0.988 years.

The method developed in this paper addresses and proposes solutions to the drawbacks in age-assessment research, which currently deals with the following:

1. Ethical issues of submitting healthy individuals to ionizing radiation for nontherapeutic purposes [10], since most of the established methods (ie, GP and TW) and recently published methods make use, mostly, of radiographs as the analysis input [23]. This paper showed that it is possible to achieve a good estimation of age by employing MRI images instead.
2. Lowering the risk of intra- and interrater variability, which can be very high when general radiologists are employed in the assessment of age instead of high-expertise pediatric radiologists [41,42]. Also, there is limited evidence that contrasts with the findings of manual raters and automatic systems regarding chronological age assessment, since most of the published material is directed to predict bone age [23]. However, a novel study reports a higher rate of false positives in classifying adults—with a threshold of 18 years—from hand images for manual raters compared to a deep learning system [43].
3. Time spent on assessment [9] addressed by the automation of the proposed method, which is able to perform evaluations in real time.

It is also important to mention that the proposed method in this paper provides the estimation of chronological age based on MRI images of the knee, contrary to most previous research, which aimed at estimating bone age and evaluating the methods using bone age and not chronological age. While the concept of bone age is certainly useful and important in many clinical settings, it was not conceived as a method to determine the chronological age of an individual. It was used to examine the developmental status of children and adolescents in comparison to their known chronological age, which can be advanced or delayed due to a multitude of factors that include chronic illnesses, hormonal disorders, etc [7,10]. The widespread use of BAA as an estimation of chronological age sometimes confuses these concepts and they are erroneously used interchangeably, as in many studies to justify the execution of BAA to judicial and civil issues. Also, it can be argued that the bone age attributed to an individual may be subjective and there is no objective way to obtain a confirmation of the exact number. In a clinical setting this may not be a problem since doctors can work with secure thresholds, but if the estimation is done for legal purposes it can become problematic, since decisions based on this estimation, especially regarding the ages of adulthood, can greatly affect the life of the individual in question.

Regarding our experiments, it is shown that for the male subjects, after the age of 20.5 the model could not identify any more information in the MRI images to discriminate the age of individuals. The same phenomenon occurred at the age of 19.5 for female subjects, which could indicate that the transformations that occur in the knee area related to the maturation process occur earlier in women than in men. This is in line with prior research on the knee region [12,24,44].

We also had satisfactory results for the problem regarding the classification of minors versus adults, considering the threshold of 18 years of age, which can be especially important in civil and judicial scenarios. Misclassification of minors as adults can often be viewed as much more problematic than the inverse, since the imputability for the application of laws, as well as guaranteed rights, may be different for these groups of individuals and usually harsher for adults. Our method can reduce that problem by distributing the errors depending on the application, using a modifiable threshold applied to the estimated age. Our method achieved an accuracy of 98.1% for male subjects and 95.0% for female subjects when it came to correctly classifying minors from the MRI images, when using a threshold that increased the accuracy for minors and sacrificed accuracy for adults.

From an operational point of view, the CNN technology employed with transfer learning can be seen as an enabler in performing research with medical images. The high cost for medical imaging can result in smaller datasets for many studies, but this caveat can be partially addressed when using the transfer learning technology on pretrained CNNs that have learned features from generic images. In this study, even if the features changed during training they were not changed much in our case. Generic features seem to work in a satisfactory way for MRI images; it is just detecting edges, corners, and blobs, which are relevant in MRI images as well as in generic images. Therefore, there is a possibility of applying automated methods even for smaller datasets. The study by Spampinato et al reported similar conclusions, but for radiographs of the hand [36].

### Comparison With Prior Work

We propose a fully automated and radiation-free method for chronological age assessment based on MRI images of the knee region, employing deep learning techniques. We could not find prior published work with the same attributes in the literature, as not much work has been done in estimating chronological age per se.

A recent study by Stern et al [43] employed MRI volumes of the hand with CNNs in order to predict chronological age of male subjects from 13 up to 19 years of age. They reported an MAE of 0.82 years for subjects under 18 years of age. They also reported results on majority age classification for male subjects between the ages of 13 and 25 years. An error of 5% for minors gave an error of 27.5% for adults, and an error of 1% for minors gave an error of 67.2% for adults. This can be compared to our results where an error of 1.9% for minors gave an error of 12% for adults on male subjects between the ages of 14 and 22 years. In an earlier study by Stern et al [45], they proposed a multi-factorial age estimation method using MRI volumes of the hand, clavicle, and teeth with CNNs. With this approach, they managed to predict chronological age of male subjects from 13 up to 25 years of age with an MAE of 1.01 years. They also reported results on majority age classification, where an error of 0.5% for minors gave an error of 25.0% for adults, and an error of 3% for minors gave an error of 18.1% for adults. This can be compared to our results, where an error of 1.9% for minors gave an error of 12% for adults on male subjects between 14 and 22 years of age. The results on majority age classification in these two papers by Stern et al [43,45] are the best published results so far, using one or multiple body parts. However, our results are significantly better even compared to their method using MRI data from three different body parts.

The study by Tang et al [46] proposed an artificial neural network model for estimating the chronological age of subjects (12-17 years old) using MRI images of the hand and wrist and other skeletal maturity factors of 79 subjects. In this study, the authors chose as the performance metric the comparison between the mean chronological age for all subjects and the mean estimated age for all subjects (ie, mean disparity), not calculating the error per subject, which could be misleading. The mean disparity measures whether there is a constant offset in the estimations, not the performance of the model on a per-subject level, like MAE does. A model can, therefore, have large errors in age estimation for all subjects and high MAE but can still have a small mean disparity; the MAE was not reported in this paper. Additionally, the reported results were on the validation set, probably due to the small sample size. In this fashion, the authors reported a mean disparity of 0.1 years between the estimated skeletal age and the chronological age.

Prior published methods for BAA that employed automated methods still focused mostly on the hand and wrist regions for the age assessment and made heavy use of radiographs as the input for their systems, as reported by a recent systematic literature review (SLR) and meta-analysis on BAA systems [23].

In this SLR, only two studies were reported to have made assessments based on the knee. The study by O’Connor et al [44] proposed a scoring system based on the assessment of knee radiographs as to the stage of epiphyseal fusion of the femur, tibia, and fibula on subjects from 9 to 19 years of age, employing regression model-building techniques. This study reported residuals of more than 2 bone-age years for both male and female individuals. The study by Fan et al [24] aimed to compare the age assessment based on the knee region from radiographs and MRI images on subjects from 11 to 25 years of age. They built regression models for bone age based on the scoring system by Krämer et al [47] for both image modalities, yielding better results for the MRI images, achieving R^2^ values (eg, the variance in the dependent variable that is predicted from the independent variables in regression models) of 0.634 and 0.654 for female and male subjects, respectively.

On the choice of medical imaging, the referred SLR reported only three studies that built systems for BAA based on MRI images; one of these was the study by Tang et al [46], mentioned previously. The study by Urchsler et al [13] designed a system with the deep learning technology to automatically locate the ossification centers on MRI images of the hand and wrist to assess the bone age of individuals, 13-20 years of age, with random forests. This study obtained an MAE of 0.850 bone-age years. The study by Hillewig et al [48] obtained MRI images from the clavicle and radiograph images from the hand and wrist of 220 subjects, 16-26 years of age, and evaluated these regions according to the Schmeling et al [49] and Kreitner et al [50] scoring systems for the clavicle and the hand and wrist, respectively. The study concluded that the assessment of the clavicle alone was not sufficient to discriminate individuals as younger or older than 18 years of age, thus requiring the information from the hand and wrist for the assessment.

Another noninvasive and radiation-free medical imaging method for the estimation of age that is reported in the literature is the assessment of retinal images, which is an approach that provides diagnostic evidence about important diseases, such as cardiovascular disease and diabetes. Retinal images were assessed with deep learning in the study by Poplin et al [51] in predicting a variety of cardiovascular risk factors, including age, which achieved an MAE of 3.26 years. Retinal images were also assessed by Ting et al [52] in estimating the prevalence and systematic risk factors for diabetic retinopathy, which included young age.

In regard to approaches that make use of deep learning methods in the field of BAA, the biggest initiative posed in recent years was done so by the Radiological Society of North America (RSNA) for the prediction of bone age: the RSNA 2018 Pediatric Bone Age Challenge [53]. This challenge aimed to encourage participants to develop algorithms that could most-accurately determine the bone age of subjects from 0 to 19 years of age, providing a database of around 12,000 radiograph images of the hand and wrist, labeled as to their bone age [53]. The participants proposed CNN models, like the ones by Iglovikov et al [54], Zhao et al [55], and Ren et al [22], which achieved MAEs of 7.52, 7.66, and 5.2 months. However good the obtained results were, they were not comparable to our results, since our aim was to predict the chronological age of a subject, and the RSNA project’s goal was to predict the bone age. It is also important to note that although these studies made use of large-enough sample sizes, the data were not uniformly distributed, as only 0.1% of the dataset was composed of individuals of 18 and 19 years of age. Additionally, Dallora et al [23] provided a meta-analysis on the performances based on seven studies, which contained all three deep learning studies mentioned previously, where the age ranges were mostly within 0-19 years of age and the performance metrics were given in MAE (bone-age months). The weighted average by the dataset size resulted in 9.96 MAE (bone-age months), which is higher than the results presented in this paper.

### Limitations

Regarding the limitations of this study, it could be argued that the sample size would not be big enough to be generalizable; therefore, we employed methods to ensure that the models did not overfit by using test sets separated from the training and validation sets. The results showed that the model was able to generalize to new data in the test sets. Additionally, further work will be directed to the collection of more data, which may improve the precision and MAE of our models.

Also, we aimed at having a uniform number of subjects for each age group, which was achieved by the data acquisition process; an exception was for the 19-year-old female subjects, who accounted for only 12 subjects, which could be seen as a caveat to the female model.

Additionally, the acquisition of ages for the first half year from each age group may interfere with the estimation accuracy of the minor versus adult classification. The largest impact occurs for the ages closest to 18 years. The missing data for those 17.5-17.99 years of age is important and we plan to collect new data to complement those ages in future work. Concerning the MAE numbers, these missing ages do not have as much impact as for the accuracy numbers.

Finally, the method was built upon data from healthy youth and young adult subjects and the effect of disorders that can affect growth was not explored.

### Conclusions

This paper proposed a model for the estimation of chronological age in youth and young adults using MRI images of the knee. Our method demonstrated good results and addressed the biggest drawbacks in the traditional age-estimation procedures that are still currently in use. Our results on majority age classification were significantly better than the best results previously published.

## Abbreviations

BAA: bone age assessment
Caffe: Convolutional Architecture for Fast Feature Embedding
CNN: convolutional neural network
EC2: Elastic Compute Cloud
GP: Greulich-Pyle
MAE: mean absolute error
MRI: magnetic resonance imaging
RSNA: Radiological Society of North America
SLR: systematic literature review
TW: Tanner-Whitehouse
VGG: Visual Geometry Group
